# Visual relations children find easy and difficult to process in figural analogies

**DOI:** 10.3389/fpsyg.2014.00827

**Published:** 2014-08-05

**Authors:** Claire E. Stevenson, Rosa A. Alberto, Max A. van den Boom, Paul A. L. de Boeck

**Affiliations:** ^1^Methodology and Statistics Unit, Department of Psychology, Leiden UniversityLeiden, Netherlands; ^2^Department of Developmental and Educational Psychology, Leiden UniversityLeiden, Netherlands; ^3^Department of Quantitative Psychology, Ohio State UniversityColumbus, OH, USA; ^4^Department of Psychology, KU LeuvenLeuven, Belgium

**Keywords:** analogical reasoning, item response theory, working memory, rule difficulty, transformation salience

## Abstract

Analogical reasoning, the ability to learn about novel phenomena by relating it to structurally similar knowledge, develops with great variability in children. Furthermore, the development of analogical reasoning coincides with greater working memory efficiency and increasing knowledge of the entities and relations present in analogy problems. In figural matrices, a classical form of analogical reasoning assessment, some features, such as color, appear easier for children to encode and infer than others, such as orientation. Yet, few studies have structurally examined differences in the difficulty of visual relations across different age-groups. This cross-sectional study of figural analogical reasoning examined which underlying rules in figural analogies were easier or more difficult for children to correctly process. School children (*N* = 1422, *M* = 7.0 years, *SD* = 21 months, range 4.5–12.5 years) were assessed in analogical reasoning using classical figural matrices and memory measures. The visual relations the children had to induce and apply concerned the features: animal, color, orientation, position, quantity and size. The role of age and memory span on the children's ability to correctly process each type of relation was examined using explanatory item response theory models. The results showed that with increasing age and/or greater memory span all visual relations were processed more accurately. The “what” visual relations animal, color, quantity and size were easiest, whereas the “where” relations orientation and position were most difficult. However, the “where” visual relations became relatively easier with age and increased memory efficiency. The implications are discussed in terms of the development of visual processing in object recognition vs. position and motion encoding in the ventral (“what”) and dorsal (“where”) pathways respectively.

## Introduction

Analogical reasoning is considered a central feature of human cognition that is essential to learning (Alexander et al., 1989; Goswami, 1991) and develops with great variability throughout childhood (Siegler and Svetina, 2002; Tunteler et al., 2008). When solving an analogy a known concept or situation is applied to a new comparable situation. A number of processes are involved in analogical reasoning, such as identifying relations, keeping multiple relations in memory and manipulating them to formulate an answer (Sternberg, 1977; Mulholland et al., 1980; Goswami, 1991; Hummel and Holyoak, 2005). Complexity of analogy problems have often been related to dimensionality (Halford et al., 1998; Halford and McCredden, 1998; Andrews and Halford, 2002) and the number of relations to be processed (Mulholland et al., 1980). The more complex an analogy is the greater the requirements for processing and storage capacity and efficiency—working memory components that increase with age (Fry and Hale, 2000; Kail, 2007; Swanson, 2008). Indeed, recent research on the development of analogical reasoning explains age-related improvement in terms of enhanced executive functioning, with a focus on improved inhibition control and increased working memory efficiency (Richland et al., 2004; Thibaut et al., 2008). However, the type of relations involved in an analogy may require further consideration in the discussion of complexity as some features, such as color, appear easier for children to encode and infer than others, such as orientation (e.g., Siegler and Svetina, 2002; Stevenson et al., 2011). A few studies have examined the difficulty of rule-types (Carpenter et al., 1990) and perceptual features (Meo et al., 2007) in matrix analogies such as Raven's figural matrices; yet, these have not yet been examined from a developmental perspective. This cross-sectional study of elementary school children's figural analogical reasoning aims to provide a structural examination of the difficulty of visual relations in relation to differences in age and working memory.

Halford and colleagues (Halford et al., 1998; Halford and McCredden, 1998; Andrews and Halford, 2002) defined conceptual complexity analogies in terms of dimensionality: the number of entities that are related and as such should be processed in parallel. A binary relation is the relationship between two arguments, for example LARGER (mouse, elephant). Similarly, ternary and quaternary relations hold three and four relations respectively. The proposed complexity metric states that as more entities are dependent upon each other the greater the dimensionality, complexity and most importantly processing load and storage capacity required. In the present study only binary relations are used which are considered to be able to be processed from approximately 2 years of age (Halford et al., 1998). Although children differ in their ability to solve these binary relations, the model does not account for differences in intrinsic complexity in the various types of binary relations (such as larger vs. darker or position and so on).

Similarly, Mulholland et al. (1980) computed item difficulty of classical A:B::C:D analogies in terms of the number of entities in A and B multiplied by the number of rules that need to be applied to these entities to obtain the solution D. This metric has been effectively used to predict which items are easier or more difficult for children to process, in some cases explaining approximately 80% of variance in the correct solving of figural analogies (Hosenfeld et al., 1997; Stevenson et al., 2013b). However, there is some evidence that not all visual relations are equally well processed by different age groups. For example, Inhelder and Piaget (1964), one of the first to study the development of matrix completion, observed that it was easier for children to choose the appropriate object, size and color than the correct orientation. Siegler and Svetina (2002) found that object and color were processed correctly more often by 6 year olds than orientation and size. Furthermore, Stevenson et al. (2011) demonstrated that the object and color features were easier for 4–5 year old children to process in figural matrix analogies, followed by size and orientation, while quantity and position appeared to be most difficult.

The differences Inhelder and Piaget (1964) found with regard to processing difficulty have been replicated and assessed in terms of perceptual salience (Odom and Corbin, 1973; Odom et al., 1975; Aschkenasy and Odom, 1982). Perceptual salience can be defined as the degree to which the perceptual system is sensitive to information. Odom et al. (1975) categorized the visual relations object and size as more salient and orientation as less salient. West and Odom (1979) found that saliency, measured by a location recall task, could be increased by merely exposing children to practice cards containing the low salient dimensions—an experience that resembles everyday live exposure to objects and relations. Saliency was not further enhanced by requesting a more elaborate explanation of the relations in the task. Thus, West and Odom (1979) explained these effects in terms of increased perceptual experience, something which occurs naturally with age.

In addition to increased experience with age, neurological maturation may also explain differences in salience in various visual relations between analogy entities. There is a distinct parallel between the more and less salient types of relations and neurological visual processing of the ventral and dorsal paths. Whereas the ventral pathway processes visual identity and feature information (e.g., color, luminance, faces and object identities), the dorsal pathway processes spatial relations and direction of motion information. Visual relations in analogies can be categorized under object identification and recognition (highly salient) vs. spatial features (less salient). These are processed in parallel yet separate visual pathways—the (magnocellular) dorsal “where” pathway and (parvocellular) ventral “what” pathway (Spencer et al., 2000; Parrish et al., 2005; Taylor et al., 2009). In typically developing children the maturation of the dorsal stream appears to be more prolonged in comparison to the ventral stream (Gunn et al., 2002; Braddick et al., 2003; Mitchell and Neville, 2004; Parrish et al., 2005). For example, Gunn et al. (2002) compared performance of 4–11 year olds and adults on visual processing tasks involving form and motion. The form and motion tasks showed similar developmental trends. Although, the coherence threshold did not differ between the youngest age group and the adults on tasks involving form; however, motion coherence thresholds on the motion task reached adult levels at a later stage, 10–11 years of age. These findings imply that the correct processing of motion related features in the dorsal “where” pathway may develop later than the “what” features.

Siegler and Svetina (2002) found no age related differences in 6–7 year olds among the relative improvement of correct processing for the color, object, size and orientation features indicating the relative difficultly of visual relations was stable within this age-group across a six session training phase. However, given the results of Gunn et al. (2002) we expect that the age range in the present study, 5–11 years, is broad enough to observe differential improvement with increasing age between both more salient “what” and less salient “where” visual relations.

Analogical reasoning abilities not only appear to improve with age, but also through increases in working memory efficiency and cognitive control (Richland et al., 2006; Thibaut et al., 2010). Studies have shown a strong relationship between working memory efficiency and children's reasoning ability (Alloway et al., 2004; Tillman et al., 2008; Stevenson et al., 2013a). Improvement in working memory seems to coincide with improvement in reasoning and problem solving in children (Fry and Hale, 2000; Kail, 2007; Swanson, 2008). Meo et al. (2007) suggested that element saliency facilitates an efficient representation in working memory. These economically beneficial representations of salient visual relations are expected to place a smaller load on working memory; thus more salient visual relations would tax working memory less. In the current study, it was therefore expected that working memory capacity would be related to the correct processing of all types of visual relations. In the light of prolonged development of the dorsal vs. ventral stream it was further hypothesized that greater working memory would be especially beneficial for the processing of dorsal stream “where” visual relations.

The current study examined the relative difficulty of different visual relations for children of different ages and working memory capabilities. It was hypothesized that the visual relations, object, color, size and quantity, processed by the ventral “what” stream would be more likely to be solved correctly than orientation and position, visual relations processed by the dorsal “where” stream. Greater working memory efficiency and increased age were expected to positively influence the solving of all visual relations. Furthermore, in line with the prolonged maturation of the dorsal stream, the relative difficulties of the “where” vs. “what” relations were expected to lessen with age and greater memory span.

## Materials and methods

### Sample

In total 1422 children aged 4–12 years (47–151 months) were included in this study. The sample was a collection of participants from 8 experiments conducted between 2009 and 2012. The sample consisted of 590 boys and 832 girls, with a mean age of 6.99 years (*SD* = 1.75), 89.43 months (*SD* = 20.86). Written informed consent for children's participation was obtained from the parents prior to data collection.

### Design and procedure

Each child was tested individually in a quiet room at the child's school by a psychology student trained in the procedure. The instructions were provided according to standardized protocols. All children were administered the figural analogy task (described in section The figural analogy task) and an age appropriate working memory task (described in section Memory tasks).

### Measures

#### The figural analogy task

**Figure 1:**
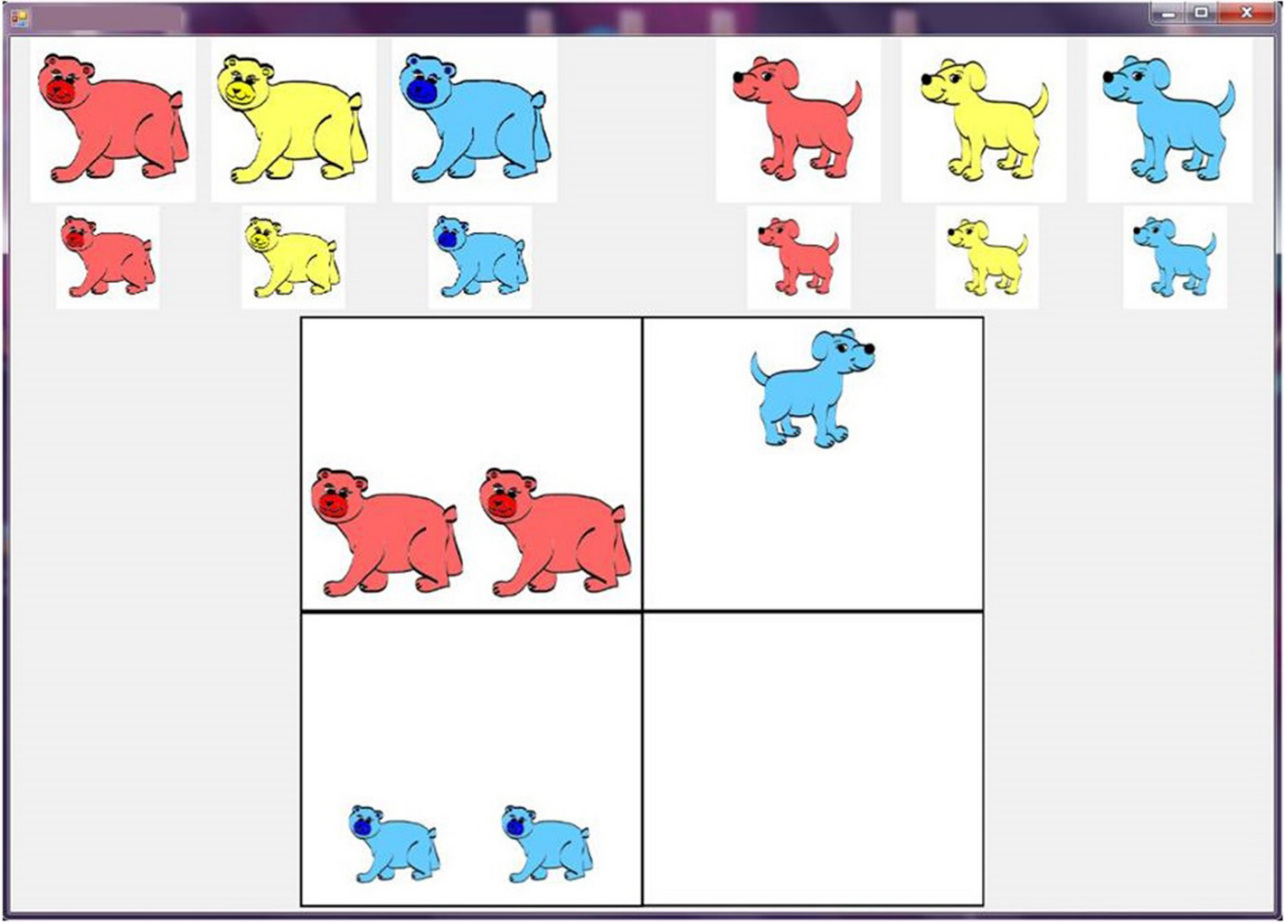
**Example of a figural analogy matrix**. The matrix contains seven visual relations (horizontal: animal, color, orientation, position, quantity; vertical: color and size).

The figural analogies (A:B::C:D) comprised of 2 × 2 matrices with familiar animals as objects. The animals changed horizontally or vertically by animal-type, color, orientation, size, position and quantity (see Figure 1). The number of visual relations (range: 2–8 visual relations; *M* = 4.87, *SD* = 1.99) were equally distributed across the 71 items. The children had to construct the solution using a computer mouse to drag and drop animal figures representing the six visual relations into the empty box in the lower left or right quadrant of the matrix. A maximum of two animals were present in each analogy. These were available in three colors (red, yellow, blue) and two sizes (large, small). The orientation (facing left or right) could be changed by right-clicking the animal in the answer cell. Quantity (one or two animals) could be specified by allowing the child to drag and drop multiple animals in the answer cell. Position was specified by location of the animal placed in the answer cell (top, middle or bottom).

#### Memory tasks

Memory was assessed using different instruments during different experiments. The WISC-III digit span was administered to 6–9 year old children, the RAKIT memory span to 4–6 year olds and the Dutch version of the AWMA to 5–12 year old children. In order to be able to compare the different measures of working memory, raw memory scores from the different tasks were converted into norm scores per task and then standardized to one common z-score. The standardized (z-) scores were used in our statistical analyses.

***WISC III digit span***. The Wechsler Intelligence Scale for Children (WISC-III) Digit Span subtest measures the verbal memory span and manipulation capacity for children from 5 to 15 years. The child is presented with a list of spoken digits at a rate of approximately one digit per second and is asked to recall the digits in either forward or backward order. The digit span is based on length of the longest sequence of digits that the child could recall correctly 2 times in a row.

***RAKIT memory span***. The Revised Amsterdam Children's Intelligence Test (RAKIT, Bleichrodt et al., 1987) memory span subtest measures concrete and abstract visual memory of children from 4 to 11 years old. The child is shown a large card with a series of concrete or abstract figures. The card is then is removed and the child must reproduce the figures series in the same forward order using a set of cards containing the same figures.

***AWMA Listening recall and Spatial span***. The Automated Working Memory Assessment (AWMA, Alloway, 2007) is used to measure the working memory in children and young adults from 4 to 22 years old. Two subtests of the AWMA were used: the Spatial Span subtest to assess visuo-spatial working memory and the Listening Recall subtest to assess verbal working memory. The Spatial Span subtest presents a sequence of two figures and the child is asked to say whether these are facing the same or the opposite direction of each other. After the entire sequence the child must recall the order where the red dots were located in each of the figures. The Listening Recall subtest is verbal working memory subtest which consists of spoken sentences, where the child is asked to say whether the sentence is true or false (e.g., bicycles can walk). Additionally, after the sequence of sentences, the child must repeat the first word of each sentence in order. The mean of these two AWMA subtests was used to represent the WM score in this study.

### Statistical analyses

Our main research aim was to investigate which visual relations children of different ages and levels of working efficiency find easier and more difficult to process. This was investigated using explanatory item response theory (IRT) models. Explanatory IRT can be seen as multilevel (items nested within persons) binary logistic regression. The base model is a simple IRT model with random intercepts for both persons and items where the probability of a correct response of person *p* on item *i* is expressed as follows.

(1) $$\begin{array}{l} P(y_{pi}=1|\theta_{p},\beta_{i})=\frac{\exp(\theta_{p}-\beta_{i})}{1+\exp(\theta_{p}-\beta_{i})}\\ \text{where }\theta_{\text{p}}\sim \text{N}(0,\sigma_{\theta }^{2})\text{ and }\beta_{\text{i}}\sim \text{N}(0,\sigma_{\beta }^{2}) \end{array}$$

Our base model assumes both persons and items to be random variables (Baayen et al., 2008; De Boeck, 2008).

We added an additional level to this base model in order to specifically investigate children's performance per transformation feature (animal, color, orientation, position, quantity and size). Thus, in the present context the correctness per transformation feature is the dependent variable. This is an item response theory model where the probability of a correct response of person *p* on item *i* for transformation *r* is expressed as follows:

(2) $$\begin{array}{l} P(Y_{pir}^{*}=y_{pir}^{*}|\theta_{p},{\underline{\beta }}_{i})=\frac{\exp{\left(\theta_{p}-\beta_{ir}\right)}^{y_{pir}^{*}}}{1+\exp(\theta_{p}-\beta_{ir})}\\ \text{where }\theta_{\text{p}}\sim \text{N}(\mu ,\sigma_{\theta }^{2})\text{ and }\beta_{\text{i}}\sim \text{N}(\mu ,\sigma_{\beta }^{2}) \end{array}$$

We worked with three levels where transformation features are nested within items, and items nested within children (e.g., De Boeck and Partchev, 2012).

This item response model was then extended with explanatory variables in order to evaluate their effects on the latent scale of analogical reasoning ability (e.g., De Boeck and Wilson, 2004; Stevenson et al., 2013b). Both person predictors such as age and working memory as well as the item predictor transformation type were included to explain the children's performance on the figural analogies scale. The models were estimated using the lme4 package for R (Bates and Maecheler, 2010).

## Results

The aim of this paper was to determine which visual relations, “what” vs. “where,” children find more difficult and easy to process while accounting for differences in item difficulty. Furthermore we investigate the roles of age and working memory on children's processing of the different visual relations. Prior to conducting analyses initial comparisons of subject characteristics and psychometric properties were assessed.

### Initial comparisons

As can be seen in Table 1 the processing of each visual relation generally improved with age. Furthermore, on the whole, the animal transformation was solved correctly more often than other transformation types, followed by color and size. In contrast, the position transformation seems to be the most difficult transformation. Correlations between working memory and proportion correct per transformation (without taking item difficulty into account) were all significant (*p* < 0.001) and ranged from weak for size (*r* = 0.26), moderate for quantity and orientation (*r* = 0.32 and *r* = 0.33 respectively) and strong for animal, position and orientation (*r* = 0.41, *r* = 0.42 and *r* = 0.44 respectively). The ability to solve each of the separate visual relations were all strongly related (*p* < 0.001; see Table 2).

**Table 1:** **Proportion correct solutions for each visual relation by age group**.

**Age (Years)**	**Animal *M* (*SD*)**	**Color *M* (*SD*)**	**Orientation *M* (*SD*)**	**Position *M* (*SD*)**	**Quantity *M* (*SD*)**	**Size *M* (*SD*)**
5–11	0.73 (0.22)	0.66 (0.20)	0.59 (0.22)	0.53 (0.24)	0.62 (0.20)	0.65 (0.21)
5	0.62 (0.19)	0.56 (0.17)	0.50 (0.18)	0.40 (0.19)	0.49 (0.16)	0.53 (0.20)
6	0.64 (0.21)	0.57 (0.18)	0.50 (0.19)	0.43 (0.22)	0.53 (0.18)	0.57 (0.18)
7	0.68 (0.21)	0.63 (0.19)	0.53 (0.20)	0.47 (0.23)	0.57 (0.20)	0.61 (0.19)
8	0.76 (0.22)	0.71 (20)	0.60 (0.22)	0.58 (0.23)	0.68 (0.21)	0.70 (0.19)
9	0.87 (0.15)	0.76 (0.18)	0.75 (0.20)	0.69 (0.20)	0.75 (0.17)	0.78 (0.17)
10	0.90 (0.11)	0.83 (0.14)	0.79 (0.17)	0.73 (0.18)	0.76 (0.15)	0.82 (0.15)
11	0.90 (0.12)	0.81 (0.16)	0.74 (0.19)	0.72 (0.21)	0.75 (0.19)	0.78 (0.16)

**Table 2:** **Correlations between proportion correct solutions for each visual relation**.

	**Animal**	**Color**	**Orientation**	**Position**	**Quantity**	**Size**
Animal	1	0.58	0.59	0.58	0.53	0.56
Color		1	0.49	0.53	0.57	0.55
Orientation			1	0.54	0.51	0.47
Position				1	0.57	0.50
Quantity					1	0.55
Size						1

### Psychometric properties

Cronbach's alpha coefficient of internal consistency ranged from 0.70 to 90 for the different analogy test versions. The reliabilities were all considered satisfactory. The proportion correct responses per item ranged from 0.01 to 0.80 (*M* = 0.25, *SD* = 0.19). The rank correlation between the proportion incorrect and the predicted difficulty level based on the number of visual relations was *r* = 0.53, *p* < 0.001, indicating that as the number of visual relations increased the proportion correct solutions decreased.

### Difficulty of visual relations by age and working memory

The dependent variable was the correct/incorrect solution per visual relation per item. To account for correlations in performance per transformation within items and also items within persons item response analysis was applied (see Section Statistical analyses). The following hypotheses were tested with this model: (1) “what” relations are more likely to be solved correctly than “where” transformations, (2) the chance of solving each of the relations correctly increases with age, (3) children with higher working memory scores are more likely to correctly solve each of the of relations than children with lower working memory scores, (4) the relative difference in difficulty between “what” and “where” relations is greater in younger children than older children and also (5) there is a relatively larger difference in the ability to solve “what” vs. “where” relations for children with lower memory scores than children with higher memory scores. Contrast coding of “what” vs. “where” transformations were used to aid in testing these hypotheses: “what” transformations animal, color, size and quantity were coded with 1; “where” transformations orientation and position were coded with –1. Based on the literature and initial comparisons of the separate visual relations (see Section Initial comparisons) this distinction was considered warranted.

The results of the model used to investigate these hypotheses are shown in Table 3. Random intercepts were present for persons (*SD* = 0.31) and items (*SD* = 0.50). A main effect was found for the “what” vs. “where” relations contrast: *B* = 0.13, *SE* = 0.01, *p* < 0.001, indicating that the odds of solving a “what” transformation correctly was 1.14 times more likely than correctly solving a “where” transformation. The significant main effect of age (*B* = 0.29, *SE* = 0.01, *p* < 0.001) indicated that the chance that a child solved a transformation within an item correctly increased by 0.29 logits (odds 1.33) per one standard deviation (1.75 years) increase in age. The resulting probability of correctly solving a transformation was 0.42 for 5 year olds, 0.50 for 7 year olds, 0.58 for 9 year olds and 0.66 for 77 year olds. The significant main effect of working memory (*B* = 0.17, *SE* = 0.01, *p* < 0.001) showed that the odds of solving a transformation within an item was 1.19 times greater per one standard deviation increase in working memory. This translates to a probability of 0.41, 0.50, 0.59 for children with low (−2 *SD*), average and high (+2 *SD*) working memory scores. Thus, increased age and greater working memory both made correct processing of the different visual relations within an item more likely. Furthermore, there was a marginal Age × Working Memory interaction (*B* = 0.03, *SE* = 0.01, *p* = 0.06), indicating a slight additional positive effect on correct analogy solving for older children with higher working memory scores.

**Table 3:** **Estimates of fixed effects of explanatory item response model predicting chance of correct solution for “what” vs. “where” transformations by age and working memory score**.

	**B**	**SE**	**Lower Bound (95% CI)**	**Upper Bound (95% CI)**	***Z***	***P***
Intercept	−0.12	0.09	−0.21	−0.04	−1.41	0.16
What vs. Where	0.13	0.01	0.13	0.14	17.56	<0.001
Age	0.29	0.01	0.27	0.30	19.36	<0.001
Working memory	0.17	0.02	0.16	0.19	11.21	<0.001
Age × Working memory	0.03	0.01	0.01	0.04	1.92	0.06
What vs. Where × Age	−0.03	0.01	−0.04	−0.02	−3.48	<0.001
What vs. Where × Working memory	−0.04	0.01	−0.05	−0.04	−5.21	<0.001
What vs. Where × Age × Working memory	0.01	0.01	0.00	0.02	1.46	0.15

*Note. Age and working memory were included in the model as z-scores*.

**Figure 2:**
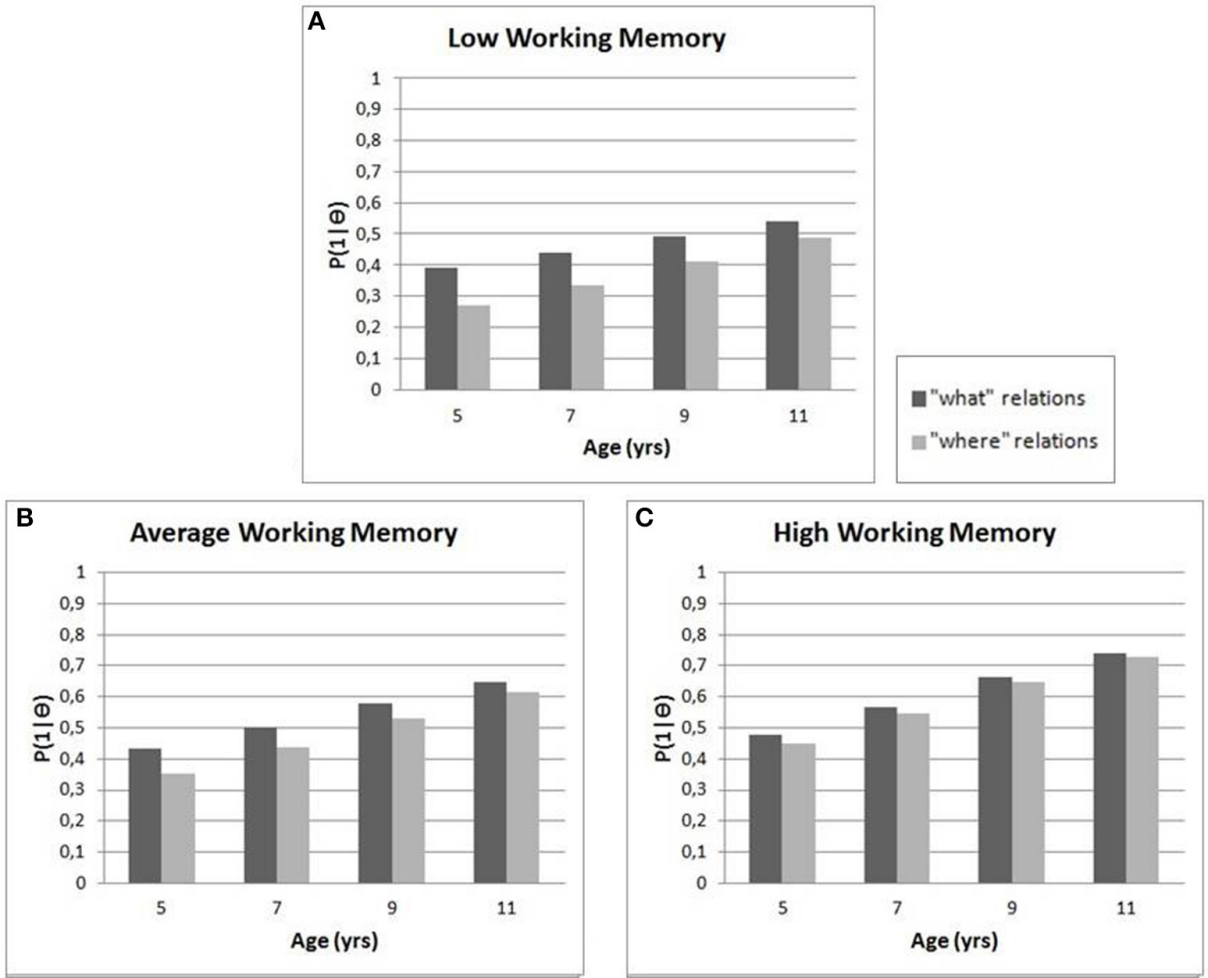
**Processing of visual relations for children of different ages with (A) low, (B) average and (C) high working memory scores**. Correct solutions for all visual relations improved with age and greater memory skills. “What” visual relations (animal, color, size and quantity) were easier for children than “where” relations (orientation and position); however, the gap between “what” and “where” visual relations decreased with age and greater memory skills.

To further investigate the effect of Working Memory (WM) and Age on the “what” vs. “where” visual relations two- and three-way interaction effects were included in the model. The results are depicted in Figure 2. There was a significant interaction effect between the “what” vs. “where” relations contrast and Age: *B* = −0.03, *SE* = 0.01, *p* < 0.001. The chance of a correct solution for the “where” relations increased more with age (while holding working memory score constant) than the chance of correctly solving a “what” relation. More specifically, the odds of correctly solving a “where” transformation was 1.03 higher per standard deviation of age (1.75 years) compared to solving a “what” transformation correctly. The effect of WM on the “what” vs. “where” transformations (while holding age constant) was also significant: *B* = −0.04, *SE* = 0.01, *p* < 0.001. The negative effect here shows “where” transformations were easier for children with higher working memory scores than children with lower working memory scores (odds ratio 1.04). For example, for an average ability child on an item of average difficulty, the chance of solving a “where” transformation was 0.33 vs. 0.44 for a “what” transformation if the child had a low WM score (−2 *SD*). In contrast, if the child with a high WM score (+2 *SD*) the gap was smaller with a chance of 0.55 for a “where” transformation and 0.57 for a “what” transformation. The interaction effect of What-Where × Age × WM was not significant (*B* = 0.01, *SE* = 0.01, *p* = 0.15).

In sum, the chance of correctly solving “where” transformations improved substantially more with age and level of working memory than for “what” transformations. However, “what” transformations were easier for children of all ages to solve correctly that “where” transformations.

## Discussion

The aim of this cross-sectional study of figural analogical reasoning was to assess which visual relations were easier or more difficult to solve for children of different ages and memory skills. The three main findings were: (1) the difficulty of visual relations in analogies for children appears to be related to the differential development of dorsal “what” and ventral “where” visual processing pathways; (2) the relative difficulty of “what” vs. “where” relations decreases with age; and (3) after correcting for the effects of age the relative difficulty of “what” vs. “where” relations is less profound in children with greater working memory efficiency. The discussion is ordered along the lines of these main findings.

### Visual relations: “what” vs. “where” features

As expected we found that the visual relations processed by the ventral “what” stream, object, color, quantity and size, were easier for children to solve that the visual relations, orientation and position, processed by the dorsal “where” stream. Interestingly, these results coincide with earlier studies explaining differences in analogy rule difficulty based on saliency (Odom et al., 1975; Stevenson et al., 2009; Pronk, 2013), i.e., highly salient relations appear to coincide with “what” features and are easier for children to process than low salient relations such as position and orientation that fall under the “where” stream.

Our results are in line with Pronk (2013) who included a training of analogical reasoning in her design and found the same distinction between the different types of visual relations. Furthermore, accuracy and the ability to verbally report the relevant relations improved over time, although this differed based on the type of training received. Children who only practiced solving figural analogies on multiple occasions and were asked to verbalize how they solved the analogies, referred to the “what” relations, color, size and quantity more often than the “where” relations orientation and position. In contrast, children who also received an intensive training session mentioned all visual relations more frequently and referenced the more difficult visual relations orientation and position (Stevenson, 2012; Pronk, 2013). These results imply that visual relations in figural analogies differ in difficulty and suggest that training may have a positive effect on the visual processing of analogies through perceptual and conceptual experience.

Further support for the “what” vs. “where” distinction in visual processing may come from developmental psychology research. For example, on the balance beam task, in which children have to consider both weight and distance from the fulcrum to determine which way the scale will tip, children tend to reason by weight (i.e., size), a “what” feature, at an earlier stage in development than distance (i.e., position), a “where” feature when solving the balance beam task before finally integrating both features (Siegler and Chen, 1998). Research in developmental disabilities also appears to support the “what” vs. “where” feature distinction and their differences in difficulty of processing. For example, impaired motion processing, but not for object form, has been shown in autism (Spencer et al., 2000), dyslexia (Hansen et al., 2001) and preterm children (Taylor et al., 2009).

### Role of age and working memory

Increased age and a more efficient working memory have a positive influence on children's ability to encode, map and apply visual relations in analogy solving. Through increased exposure over time to various visual relations and representations, perceptual experience naturally increases with age; thus saliency can also be expected to increase. Increased saliency through perceptual experience would be expected to (a) follow a steady increase and (b) similarly influence the processing of different visual relations. However, our findings indicate that differential development in the perception of the different visual relations is more likely.

Our results show an interaction effect where younger children and children with a less efficient working memory have more difficulty with “where” relations relative to “what” relations. The improvement was relatively steady for “what” relations, whereas the growth in “where” relations was the most substantial between the ages of seven and nine. This may be explained by the differences in development of the ventral “what” (e.g., shape, color, size) and the dorsal “where” pathway (Braddick et al., 2000; Gunn et al., 2002; Parrish et al., 2005; Rice et al., 2007). Later maturation of the dorsal pathway may explain why the motion related visual relations, orientation and position, were generally more often solved correctly by older children than younger children. The change we found in relative difficulty of the different visual relations around seven to eight years coincides with the age range in which a “relational shift” in analogical reasoning, where children change from a focus on superficial perceptual features to reasoning about the relations between the elements in the analogy, has been found to occur (Gentner, 1988). A shift in analogy solving ability has been found in different forms of analogical reasoning, for example with scene analogies (Richland et al., 2006) as well as visual analogies comprising similar visual relations to those in the present study (Hosenfeld et al., 1997). The relational shift is proposed to occur at different ages in different domains due to increasing domain knowledge (Rattermann and Gentner, 1998) and decreasing maturational limitations in processing capacity (Richland et al., 2006) with age. Although an abrupt shift may be an artifact of a cross-sectional design, gradual changes in relative difficulty of the different relations are proposed to occur and perhaps for some rule-types this “shift” takes place earlier on in development than for others.

However, our results with regard to differences in relative difficulty between the “what” and “where” visual relations with age are perhaps in contrast with the finding of Siegler and Svetina (2002), where although all relations were easier for older children, the visual relations color, object, size and orientation were found to be of relatively similar difficulty within both of the groups they studied (6 year olds and 8 year olds). However, the older children in the Siegler and Svetina (2002) study were better capable of verbally reporting which changes they had applied in the analogies; and orientation was reported relatively more commonly in 8 year olds than 6 year olds. Furthermore, the discrepancy between their study and the present findings could perhaps be explained by the greater variance and range of ages in participants in the current study.

Richland et al. (2006) considered executive functions limitations rather than lack of domain knowledge an underlying factor in the shift from reasoning about perceptual elements to relational elements. Possibly the dorsal / ventral pathways play a role here as well. In this case a greater effect of working memory on the processing of “where” visual relations would be expected. Indeed, our findings show that the visual relations orientation and position were solved better by children with higher working memory scores while controlling for age differences.

### Limitations and future directions

A few limitations must be considered when drawing conclusions about the development in children's processing of visual relations in figural analogies based on these results. First, the study design was cross-sectional rather than longitudinal, which limits the reliability of age-related changes. A microgenetic or training study would reveal more fine-grained information on the actual development in relative difficulty of visual relations in figural analogies.

Second, we proposed that the difficulty of the visual relations to be related to the maturation of ventral and dorsal neuronal pathways (Gunn et al., 2002; Braddick et al., 2003; Mitchell and Neville, 2004; Parrish et al., 2005) without having measured the neural correlates in children while processing these relations. It would be of particular interest to test whether the observed differences in the processing of “what” and “where” are indeed a consequence of differential processing by dorsal and ventral streams. Future research could focus on cross-sectional or training-related neuronal changes using *f*MRI (e.g., Kroger et al., 2002; Bunge et al., 2003). This most likely will require a different item paradigm in which “what” and “where”-type relations are not combined in items, or perhaps an examination of each visual relation presented separately as the mere presence of “where” relations in an otherwise easy analogy may influence the processing of additional “what” relations and vice versa.

Third, not all children were administered the same memory measure. The measures we used may not all measure the same aspects of (working) memory. Indeed, we used a mixture of visual and verbal memory measures, although these may be considered separable in children (Alloway et al., 2006) and may influence the extent to which memory was related to the solving of the visual relations. Although based on the established relationships between both visual and verbal memory and analogical reasoning in children (e.g., Stevenson et al., 2013a) we can conclude, despite this limitation, that memory is related to the difficulty of processing both “what” and “where” features. In future research we recommend administering working memory tasks in both visual and verbal format to all participants.

Finally, an alternative interpretation for the role of working memory in which visual relations children found easiest to solve could be related to the order in which the visual relations were processed while solving the analogies. In the analogies used in present study (Figure 1) animal, color and size are most likely determined prior to selecting an analogy figure, whereas orientation, position and quantity are probably determined while placing the figure in the solution box; thus the visual relations specified last, were perhaps most easily forgotten. A study that examines the sequence mouse events and eye movements would help clarify this. However, this does not explain why quantity which must be applied later and requires additional effort in the form of a drag and drop movement across the screen was easier than orientation (click only) and size (short distance drag and drop). We can conclude that a more efficient working memory is an asset in solving figural analogies—especially those containing the visual relations animal, orientation and position. Therefore, further investigations could focus on improving performance on the motion related visual relations in young children and thereby enhance general performance on figural analogy problems. These results might be implemented in educational settings. Owing to the proposed dorsal pathway plasticity (Mitchell and Neville, 2004; Maurer et al., 2005), children might have a substantial added benefit when provided training in “where” relations which could be assessed in a longitudinal manner utilizing a pretest-training-posttest design.

## Conclusions

In sum, this cross-sectional study of figural analogical reasoning demonstrates that the difficulty of visual relations in analogies for children can be theoretically related to the differential development of dorsal “what” and ventral “where” visual processing pathways, in which the relative difficulty of “what” vs. “where” relations decreases with increasing age and working memory capacity. Further research should focus on directly assessing the neural correlates of the processing of “what” and “where” transformations.

### Conflict of interest statement

The authors declare that the research was conducted in the absence of any commercial or financial relationships that could be construed as a potential conflict of interest.
